# Polyphenol-rich sugarcane extract reduces circulating *trans* fatty acids, VLDL cholesterol, and triglycerides in healthy adults

**DOI:** 10.3389/fnut.2026.1923420

**Published:** 2026-09-02

**Authors:** Simone Lewin, Claudia Hartley, Thalia Marina Llalla Vidal, Jayson J. A. Rose, Andrew Costanzo, Snehal R. Jadhav, Jacoba Bromfield, Russell S. J. Keast, Shane Mitchell, Matthew Flavel, Daniel Anthony Dias

**Affiliations:** 1 ARC Training Centre for Hyphenated Analytical Separation Technologies (HyTECH), Hobart, TAS, Australia; 2 Deakin Centre for Advanced Food Sciences, School of Exercise and Nutrition Sciences, Deakin University, Burwood, VIC, Australia; 3 Bioactives Division, The Product Makers Australia Pty Ltd., Keysborough, VIC, Australia; 4 Department of Microbiology, Anatomy, Physiology and Pharmacology, La Trobe University, Bundoora, VIC, Australia; 5 La Trobe Institute for Molecular Sciences (LIMS), La Trobe University, Bundoora, VIC, Australia; 6 Cmbio, Fruebjergvej, Copenhagen, Denmark

**Keywords:** gut microbiome, polyphenol-rich sugarcane extract, polyphenols, randomised controlled trial, trans-fatty acids, triglycerides, VLDL

## Abstract

**Background:**

Polyphenol-rich sugarcane extracts (PRSEs) are food-derived sources of naturally occurring bioactive polyphenols with increasing relevance to metabolic regulation, gut microbiome function, and overall well-being.

**Objective:**

This fully remote trial investigated the effects of PRSE supplementation on cardiometabolic biomarkers and gut microbiota.

**Methods:**

Healthy adults aged 18–55 years (*n* = 47, female = 36 and male = 11) residing in Australia were recruited to a remote quadruple-blinded, randomised, placebo-controlled cross-over trial, with each intervention arm lasting 90 days. Participants received PRSE oral capsules at 500 mg/day (two 250 mg doses) or maltodextrin placebo capsules. Blood and faecal samples were collected for subsequent blood biomarkers and microbiome analyses, respectively. Self-reported qualitative surveys were conducted to assess overall wellbeing over the 6-month period.

**Results:**

Significant treatment × time effects were observed for circulating *trans*-fatty acids (%), triglycerides and VLDL cholesterol, as well as several microbial metabolic pathways, including glycerol degradation III, pyruvate dehydrogenase, and *p*-cresol degradation. No significant effects were detected for body weight, inflammatory, or glycaemic markers following correction for multiple comparisons.

**Conclusion:**

PRSE supplementation was associated with a lower circulating proportion of *trans*-fatty acids, and lower triglyceride and VLDL cholesterol concentrations compared with placebo. PRSE supplementation also modulated selected microbial functional pathways without affecting overall microbiome diversity or community composition. These findings support the biological activity of PRSE and suggest potential interactions between host metabolic and microbiome-related mechanisms.

**Clinical trial registration:**

https://anzctr.org.au/Trial/Registration/TrialReview.aspx?id=386733&isReview=true, identifier, ANZCTR; ACTRN12624000055505.

## 1 Introduction

Cardiometabolic diseases remain a leading cause of morbidity and mortality worldwide, with risk factors including dyslipidaemia, impaired glucose regulation, chronic low-grade inflammation and obesity contributing to disease development. Dietary interventions rich in bioactive compounds, such as carotenoids, phytosterols and polyphenols, have attracted increased interest as strategies to support metabolic health (1). Among these compounds, polyphenols have received particular attention because of their broad range of biological activities and their abundance in plant-based foods (2). Polyphenols are a diverse group of plant-derived bioactive compounds that have been associated with antioxidant, anti-inflammatory, and metabolic regulatory activities. Emerging evidence suggests that polyphenol-rich foods and extracts may influence multiple aspects of host physiology, including lipid metabolism, oxidative stress, vascular function and glucose homeostasis (3).

Experimental and clinical studies have reported associations between polyphenol intake and improvements in circulating lipid profiles, including reductions in triglycerides, low-density lipoprotein cholesterol, and markers of oxidative stress and inflammation (4). However, the magnitude of benefit varies by baseline cardiometabolic status, polyphenol dose, and intervention duration (5).

In addition to their direct effects on host tissues, polyphenols interact extensively with the gut microbiome, where they undergo biotransformation and influence the production of bioactive microbial metabolites, including short-chain fatty acids (SCFAs), which contribute to intestinal barrier integrity, immune regulation, and host metabolic homeostasis (6). In turn, they may act as critical mediators linking diet, microbial activity, and host cardiometabolic function (1). Consequently, dietary interventions capable of modulating microbial activity may influence metabolic health through both direct effects on host tissues and indirect microbiome-mediated mechanisms (7).

Polyphenol-rich sugarcane extract (PRSE) is derived from sugarcane and contains a complex mixture of naturally occurring phenolic compounds (8). Experimental studies suggest that PRSE may influence metabolic health through modulation of oxidative stress, inflammatory responses, and gut microbial activity (7, 9). Given the recognised contribution of oxidative and inflammatory processes to cardiometabolic disease development, PRSE represents a promising dietary intervention for supporting cardiometabolic health (10).

Despite growing evidence from experimental studies, limited randomised controlled human trials have evaluated the effects of PRSE supplementation on cardiometabolic biomarkers and gut microbial functional pathways in humans. Therefore, the aim of the present randomised controlled trial was to investigate the effects of PRSE supplementation on cardiometabolic biomarkers and gut microbiota-associated outcomes in healthy adults. It was hypothesised that PRSE supplementation would favourably influence lipid-related cardiometabolic biomarkers and microbial functional pathways associated with metabolic regulation.

## 2 Materials and methods

### 2.1 Study design and participants

This study was conducted as a quadruple-blinded randomised, placebo-controlled crossover trial in healthy adults residing in Australia. The study was performed remotely, with participants completing all assessments and biological sample collections at home.

Participants were recruited through multiple channels, including the Deakin Centre for Advanced Food Sciences Consumer Database (HEAG-H 95_2021), flyers posted around the Deakin University Burwood Campus, print and radio media, and social media. Participants exposed to one of the recruitment strategies were prompted to complete an online screening questionnaire provided via Compusense Cloud Software (part of the Compusense Academic Consortium; v21, Compusense Inc., Guelph, ON, Canada) to determine eligibility.

Eligible participants were aged 18–55 years, fluent in English, *non*-smokers, and not pregnant, lactating, or intending to become pregnant, and consumed no more than two standard alcoholic drinks per day, with the ability to adhere to this throughout the study.

Individuals were excluded if they had a history of or current diagnosis of heart disease, hypertension, diabetes, major gastrointestinal disorders, or other chronic illnesses. Participants were also excluded if they were taking medications such as frequent antibiotics (defined as ongoing or recurrent use), *non*-steroidal anti-inflammatory drugs (NSAIDs), herbal extracts, nutrient supplements, illicit substances, or if they were involved in any other health-related clinical trial within 30 days before study commencement.

Fifty-six participants completed this study. Complete phase-specific outcome data were available for 47 participants, who were included in the final analysis. Their mean BMI was 24.49 ± 5.89 kg/m^2^, and their mean age 36.96 ± 9.02 years.

The primary outcomes considered changes in cardiometabolic biomarkers and gut microbial functional pathways. As no prior clinical data for PRSE were available in the target population, the sample size calculation was based on a prespecified standardised effect size, an accepted approach when pilot or directly comparable clinical data are unavailable (11, 12).

For the two-treatment crossover design, assuming a within-participant standard deviation of 1.0 and a treatment difference of 0.591 units, 47 participants were required to achieve 80% power at a two-sided *α* level of 0.05. This sample size was considered appropriate given the typically modest physiological effects of nutraceutical interventions in generally healthy populations. Multiple cardiometabolic endpoints were included to reflect the multifactorial and multi-target nature of nutraceutical interventions (11, 13).

**Figure 1:**
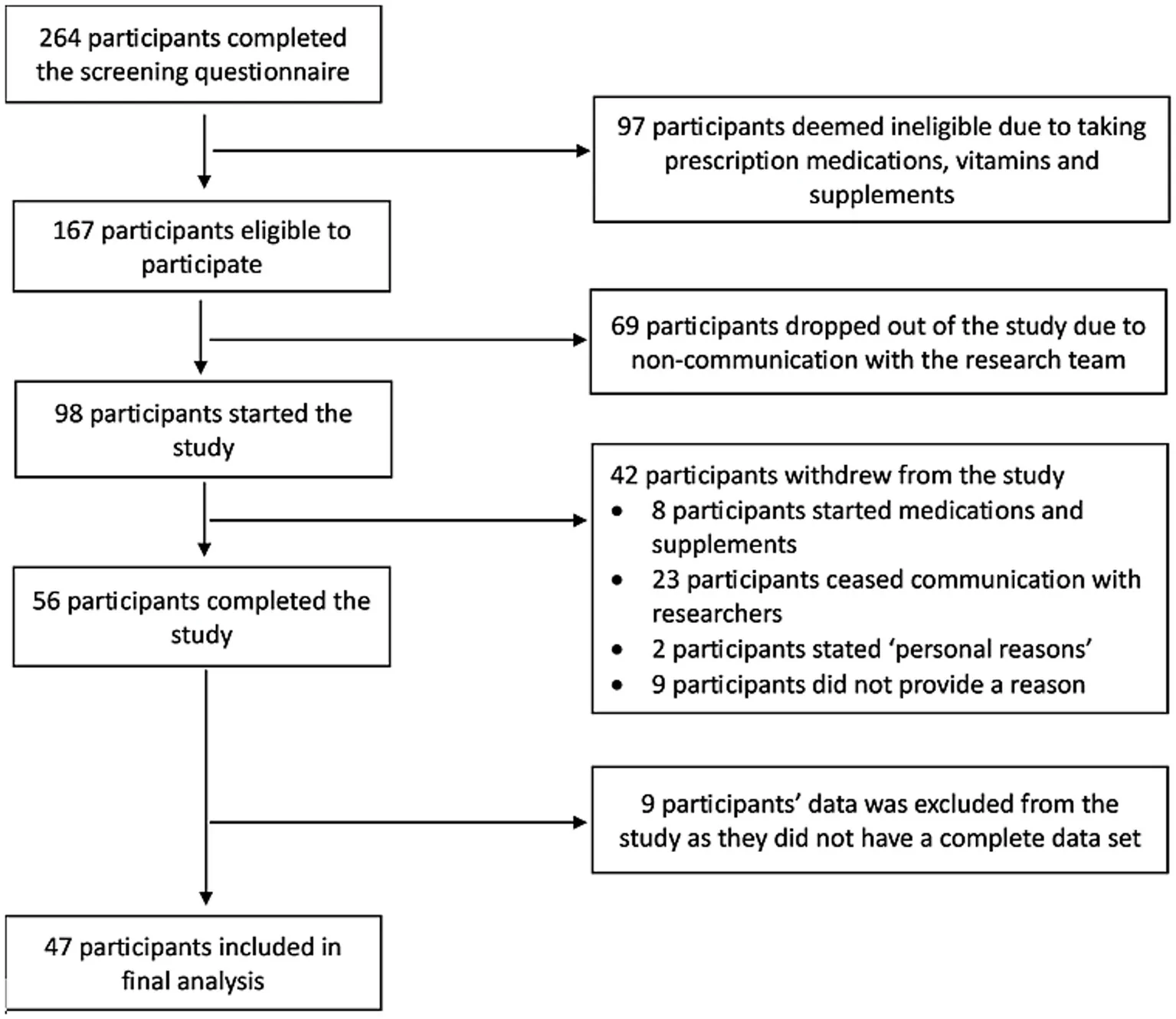
CONSORT diagram.

Nine participants who completed the study were excluded from the final analysis because one or more required outcome measurements were missing, preventing a complete within-participant crossover comparison. Additional participants were recruited but withdrew (*n* = 42) due to *non*-communication with the researcher, commencing medication or supplements, or personal reasons. See Figure 1 for a CONSORT diagram detailing participant numbers.

### 2.2 Study procedures

Participants were instructed to maintain their usual diet, lifestyle behaviours and exercise routine from 1 week prior to study commencement and throughout the study. No additional dietary interventions or behavioural modifications were introduced by the research team during the trial period.

At baseline, participants completed questionnaires assessing dietary intake and habits, mood, subjective wellbeing, demographic characteristics, medication and supplement use. Dietary intake was assessed using the Dietary Guideline Index-2013 (DGI-2013), and a modified Food Frequency Questionnaire (FFQ) specifically focusing on polyphenol intake. Mood and well-being were assessed using the Positive and Negative Affect Schedule (PANAS) and the 5-item World Health Organisation-5 (WHO-5). Participants also self-reported height and body weight, from which body mass index (BMI) was calculated.

In addition, participants provided a single morning faecal swab (Microba Life Sciences, Brisbane, Australia) and a single finger-prick blood sample (Nutripath, Melbourne, Australia). Stool consistency was assessed alongside the faecal swab using the Bristol Stool Formation Scale (BSFS).

Following baseline assessments, participants commenced the intervention (experimental group or control) for 90 days. On day 90, participants repeated all the biological sample collections and questionnaires. Participants then crossed over to the alternate condition (experimental group or control) for a further 90-day period, after which all assessments were repeated.

No additional washout period was included between intervention phases. This decision was based on the 90-day duration of each intervention period, which was considered sufficient for intervention effects to develop and be evaluated within each phase, together with the expected reversibility of the primary outcome measures after cessation of the intervention. Furthermore, because the study assessed a heterogeneous range of biomarkers with differing biological response and recovery kinetics, there is no evidence-based washout duration that would adequately address all outcomes. Previous nutrition crossover trials have similarly implemented consecutive intervention periods without a formal washout (14). While the possibility of residual carryover effects cannot be completely excluded, these were considered unlikely to materially influence the primary outcomes, and a formal washout period was therefore not incorporated into the study design (15, 16). Phase, sequence, and sequence-related interaction terms were included in the statistical models to assess systematic period and sequence effects. Nevertheless, this analysis could not conclusively exclude residual carryover effects.

**Figure 2:**
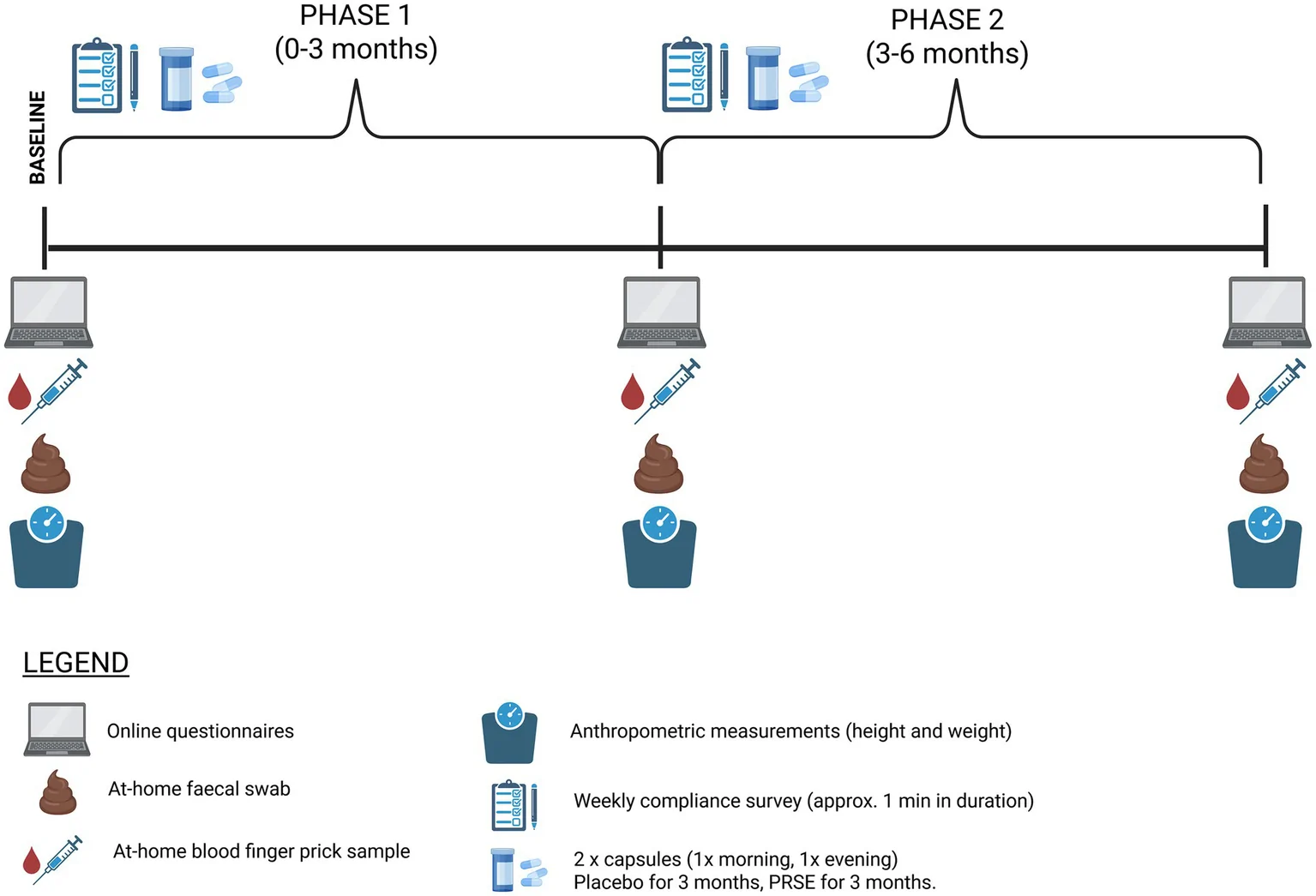
Study design of the randomised, quadruple-blinded, placebo-controlled crossover trial. Participants received either PRSE or placebo during Phase 1 (0–3 months) and crossed over to the alternate intervention during Phase 2 (3–6 months). Assessments were performed at baseline, 3 months, and 6 months.

An outline of the study design is presented in Figure 2. Participant adherence was monitored using weekly online questionnaires in which participants reported intervention capsule consumption, changes in medication or supplement use, and any adverse events.

### 2.3 Intervention

During each intervention period, participants received either 500 mg/day of PRSE (two 250 mg capsules) or matched maltodextrin placebo (two 250 mg capsules). Participants were instructed to consume one capsule in the morning and one at night, at approximately the same time each day, with or without food.

The maltodextrin placebo was colour-matched to the PRSE and encapsulated in identical gel casings to ensure blinding. The selected dose was based on findings from a Phase 1 clinical trial demonstrating its safety.

### 2.4 Randomisation and blinding

Participants were randomly allocated to one of two treatment sequences, receiving either PRSE followed by placebo or placebo followed by PRSE. Randomisation was performed using a computer-generated randomisation schedule created prior to participant recruitment. Participants were allocated sequentially according to recruitment order, with randomisation stratified by sex to ensure balanced distribution of males and females across treatment sequences.

Allocation concealment was maintained throughout the study. The manufacturer assigned a unique five-character alphanumeric code which was assigned to each treatment sequence, ensuring that participants, investigators, outcome assessors, and statisticians remained blinded to treatment allocation until all analyses had been completed.

### 2.5 Assessment of harms

Potential adverse events were monitored throughout the study using the weekly participant questionnaires and through direct participant reporting to the research team. Participants were asked to report any changes in health status, medication or supplement use. The intervention was considered low risk.

### 2.6 Outcome measures

The primary outcomes included cardiometabolic biomarkers and gut microbial functional pathways. Secondary outcomes included self-reported body weight and measures of mood and wellbeing. Diet quality was assessed as a control variable to evaluate whether dietary intake remained stable throughout the intervention period. Estimated total polyphenol intake was included as a control variable to account for natural variation in background dietary polyphenol consumption among participants.

#### 2.6.1 Cardiometabolic biomarkers

Cardiometabolic biomarkers were assessed at baseline, 90 days, and 180 days, using a finger-prick blood collection service (Nutripath, Melbourne, Australia). Biomarkers included insulin, haemoglobin A1c (HbA1c), high-sensitivity C-reactive protein (hsCRP), and lipid profile, including triglycerides, total cholesterol, high-density lipoprotein (HDL), low-density lipoprotein (LDL), very-low density lipoprotein (VLDL) and total fatty acids (saturated, monounsaturated, polyunsaturated, and trans fatty acids).

Participants conducted their own finger-prick blood sampled at home using a provided dried blood spot cards kit and returned them via registered mail the same day of collection for laboratory analysis. Blood spot samples were subsequently analysed via gas chromatography–mass spectrometry (GC–MS) to determine fatty acid composition and cardiovascular biomarkers.

#### 2.6.2 Gut microbiota

Faecal samples were collected at baseline, 90 days, and 180 days using a home collection kit (Microba Life Sciences, Brisbane, Australia) and participants posted the swabs back to Microba Life Sciences on the day of sample collection via registered mail for storage in −80 °C freezer until completion of the study.

DNA extraction was performed by Gnomix Laboratories (Bedford Park, South Australia), extracted DNA was sent to Clinical Microbiomics (Germantown, Maryland, USA) for shotgun metagenomic sequencing. Library preparation was performed using standardised commercial reagents and proprietary optimisation procedures, and sequencing was conducted using an Element AVITI at a depth of 22–52 million reads, to minimise potential technical batch effects, all samples were processed and sequenced together as a single analytical batch.

Human genomic contamination was removed by aligning raw FASTQ files to the human reference genome GRCh38.p14 using Bowtie2 (v2.4.2) and filtered to remove adapters and bases with Phred scores below 30 using AdapterRemoval (v2.3.1). Filtered paired reads with lengths ≥100 bp were retained. The abundance of each species was calculated as the mean read count normalised by effective gene length based on reads mapping to signature genes with observed read counts within the expected 99% quantile. Species abundances were set to zero if less than 5 genes with *non*-zero read counts were within the 99% quantile. Abundances were then normalised sample-wise such that the total abundance of all species sums to 100%. Microbial communities were characterised using the Clinical Microbiomics Human Microbiome Profiler (CHAMP), which generated both taxonomic and functional pathway profiles from metagenomic sequencing data. Functional profiling included annotation against the KEGG database and characterisation of Gut Metabolic Modules (GMMs) and Gut–Brain Modules (GBMs), enabling assessment of microbial metabolic capacity and neuroactive metabolic pathways (17).

#### 2.6.3 Mood and wellbeing

Perceived wellbeing and mood were assessed using the WHO-5 (18) and PANAS (19) at baseline, 90 days, and 180 days. Positive and Negative Affect Scores were calculated as the sum of their respective PANAS items.

#### 2.6.4 Dietary polyphenol intake

Polyphenol intake was estimated to control for polyphenols obtained through diet. A 45-item FFQ containing foods, beverages, or ingredients high in polyphenol content was used. The list of items high in polyphenol content was extracted from the Phenol-Explorer database (Version 3.6).

Participants reported their usual intake of each item over the previous month using a 9-response option scale ranging from “I did not eat it at all” to “6 or more servings per day.” Standard serving sizes were based on the 2013 Australian Dietary Guidelines, with corresponding weights (g) or volumes (mL) obtained from the Aus Foods 2019 database in Food Works (Xyris, version 10).

Mean total polyphenol content (TPC) per gram or millilitre for each item, determined by Folin assay, was extracted from the Phenol-Explorer database. Daily polyphenol intake was calculated by multiplying the standard serving size, reported intake frequency, and TPC for each item, then summing the values across all 45 FFQ items.

#### 2.6.5 Dietary guidelines intake

Diet quality was assessed using a 9-item questionnaire validated in US populations and adapted for Australian foods, serving sizes, and alignment with the 2013 Australian Dietary Guidelines (ADGs). Participants reported their usual intake of nine food groups over the previous month using a 9-response option scale ranging from “I did not eat it at all” to “6 or more servings per day.” The food groups included fruit, vegetables, legumes and nuts, seafood, wholegrains, refined grains, low-fat dairy, high-fat dairy and other saturated fats, and sweets/confectionery. Standard serving sizes based on the 2013 ADGs were provided as examples.

The Dietary Guideline Index (DGI) was calculated to estimate compliance with the 2013 ADGs, with a maximum score of 80. Higher scores indicated greater adherence to the ADGs and better overall diet quality. Most components were scored proportionally according to age- and sex-specific recommended intakes, while wholegrain and low-fat dairy scores were based on the proportion of total grain and dairy intake, respectively. The sweets/confectionery component was scored inversely.

### 2.7 Statistical analysis

Statistical analyses were conducted using Stata Statistical software version 16.0 (StataCorp LLC, Texas, USA) or SPSS version 30 (IBM Corp., NY, USA). Data was presented as mean ± SD unless stated otherwise. Statistical significance was accepted at *p* < 0.05. Descriptive statistics were used to describe demographic information.

To assess the effect of PRSE treatment on cardiometabolic biomarkers, linear mixed models were fitted. Fixed effects included treatment (PRSE or placebo), time (baseline and post-treatment), phase (1 or 2), sequence (PRSE → placebo or placebo → PRSE), dietary polyphenol intake, and the treatment × time, treatment × sequence, time × sequence, and treatment × time × sequence interaction terms. Participant ID was included as a random intercept to account for repeated measurements within individuals. Models were estimated using restricted maximum likelihood (REML) with Satterthwaite-adjusted degrees of freedom. Estimated marginal means, Sidak-adjusted pairwise comparisons, 95% confidence intervals, and *p*-values are reported. The treatment × time interaction was used to assess the effect of PRSE relative to placebo, while the treatment × time × sequence interaction was examined to evaluate potential carryover effects between treatment phases. Because there was no washout period included between intervention phases, the post-treatment assessment from Phase 1 served as the baseline assessment for Phase 2.

To account for multiple comparisons, false discovery rate (FDR) correction using the Benjamini–Hochberg procedure was applied within predefined families of related outcomes, including lipid profile biomarkers, wellbeing measures, and glycaemic control measures. FDR-adjusted *p*-values are reported alongside unadjusted *p*-values where applicable. Outcomes not belonging to a predefined family (e.g., body weight and hsCRP) were analysed without adjustment for multiple comparisons.

A complete per-protocol analysis was conducted, with adherence to the study defined as consumption of at least 80% of the allocated capsule, a widely used *cut-off* threshold in clinical trials (20), in which missing outcome data were not imputed.

### 2.8 Microbiome analysis

Microbiome analyses were performed using the Cosmos-Hub (Cmbio) standard microbiome comparative analysis workflow, paired with Cmbio’s CHAMP human microbiome profiler (Cmbio, Fruebjergvej 3, 2,100, Copenhagen, Denmark) (21). Samples collected at baseline, placebo (CON) and PRSE treatment periods were taxonomically and functionally profiled to characterise microbial community composition and metabolic potential.

Alpha diversity was assessed using the Chao1, Shannon, and Simpson diversity indices to evaluate microbial richness and diversity within samples. The alpha diversity analysis input metric used was normalised reads frequency, using genome-normalised number of reads to reflect the underlying microbiome community composition. Beta diversity was assessed using Bray–Curtis and Jaccard distance metrics and visualised through principal coordinate analysis (PCoA). Beta diversity reads were normalised by relative abundance.

Taxonomic and functional profiles were compared between treatment conditions using the Cosmos-Hub analytical workflows. Relative abundance heatmaps, abundance distribution plots, stacked bar plots, and diversity visualisations were generated to characterise treatment-associated microbial changes. Differential abundance analyses in gut-brain and gut metabolic functional pathways were performed using *non*-parametric statistical testing (Wilcoxon rank-sum test), with statistical significance accepted as *p < 0.05; **p < 0.01; ***p < 0.001; ****p < 0.0001.

Association between treatment and microbial taxonomic and functional profiles were evaluated using the MaAsLin3 (Multivariate Association with Linear Models) workflow (22). Total sum scaling normalisation was applied, with treatment and time point included as fixed effects and participant ID included as a random effect to account for repeated measures within the crossover design. Functional analyses included KEGG Orthology pathways, Gut Metabolic Modules (GMMs) and Gut–Brain Modules (GBMs). Where appropriate, false discovery rate (FDR) correction was applied to account for multiple testing.

## 3 Results

### 3.1 Participants

From February to August 2024, 56 participants completed this study. However, only 47 participants had complete data sets and were therefore included in the analysis. This was due to 9 participants missing data collection milestones. This final participant pool included 36 females and 11 males (Table 1).

**Table 1:** Demographic information of participants (*n* = 47).

Anthropometrics	Mean ± SD	Range
Age (years)	36.96 ± 9.02	20–53
Height (cm)	167.96 ± 8.31	153–190
Weight (kg)	69.33 ± 18.60	45.5–146.1
BMI	24.49 ± 5.89	17.13–46.64

### 3.2 Compliance

Compliance with the treatment protocol was high, with no participants falling below the predefined compliance threshold of 80%. Mean compliance was 94.7% during the placebo treatment period and 94.9% during the PRSE treatment period.

To assess whether habitual dietary patterns were maintained throughout the intervention, DGI scores were evaluated. No significant treatment × time interaction was observed for DGI (*p* = 0.697), suggesting that participants maintained a consistent diet throughout the study.

### 3.3 Cardiometabolic biomarkers

Within the lipid profile, significant treatment × time interactions were observed for triglycerides (*p* = 0.004), VLDL cholesterol (*p* = 0.004), and trans fatty acids (*p* = 0.016) which remained significant following FDR adjustment (Table 2). Triglyceride concentrations increased by 42 mg/dL and VLDL cholesterol by 8.5 mg/dL across the placebo intervention, whereas both biomarkers remained relatively stable across PRSE supplementation (−6 mg/dL and −1.0 mg/dL, respectively), indicating contrasting responses between treatments. *Trans*-fatty acid concentrations decreased by 0.07 percentage points during PRSE supplementation but increased by 0.05 percentage points during the control period. No significant treatment × time effects were observed for body weight, hsCRP, insulin, HbA1c, total cholesterol, HDL cholesterol, LDL cholesterol, saturated fatty acids, monounsaturated fatty acids, or polyunsaturated fatty acids following FDR adjustment (all *p* > 0.05).

**Table 2:** Estimated marginal means and treatment × time effects of polyphenol-rich sugarcane extract (PRSE) supplementation on body weight and cardiometabolic biomarkers.

Outcome	Treatment	Baseline	Post-treatment	Δ (95% CI)	*p*	FDR-adjusted *p*
Body Weight (kg)	Placebo	69.7	70.1	+0.5 (−0.8, 1.7)	0.677	-
	PRSE	70.1	70.3	+0.1 (−1.1, 1.3)		
hsCRP (mg/L)	Placebo	2.39	2.35	−0.04 (−0.79, 0.70)	0.756	-
	PRSE	2.29	2.41	+0.12 (−0.62, 0.86)		
Insulin (μIU/mL)	Placebo	12.4	11.8	−0.6 (−3.7, 2.6)	0.423	0.423^a^
	PRSE	11.0	12.2	+1.2 (−1.8, 4.2)		
HbA1c (%)	Placebo	4.79	4.81	+0.02 (−0.13, 0.17)	0.404	0.423^a^
	PRSE	4.75	4.86	+0.11 (−0.03, 0.26)		
Triglycerides (mg/dL)	Placebo	137	179	+42 (19, 65)	0.004	0.018^b^
	PRSE	158	153	−6 (−28, 17)		
Cholesterol (mg/dL)	Placebo	189	205	+16 (7, 25)	0.056	0.080^b^
	PRSE	200	203	+3 (−5, 12)		
HDL (mg/dL)	Placebo	48.8	53.5	+4.6 (1.3, 8.0)	0.071	0.080^b^
	PRSE	53.3	53.6	+0.3 (−3.0, 3.6)		
LDL (mg/dL)	Placebo	112	115	+3 (−6, 12)	0.873	0.873^b^
	PRSE	115	119	+4 (−5, 13)		
VLDL (mg/dL)	Placebo	27.4	35.8	+8.5 (3.9, 13.1)	0.004	0.018^b^
	PRSE	31.6	30.6	−1.0 (−5.5, 3.5)		
Saturated Fatty Acids (%of total fatty acids)	Placebo	35.7	35.9	+0.2 (−0.6, 0.9)	0.775	0.873^b^
	PRSE	35.6	35.9	+0.3 (−0.4, 1.1)		
Monounsaturated Fatty Acids (% of total fatty acids)	Placebo	22.1	24.3	+2.2 (1.2, 3.2)	0.027	0.063^b^
	PRSE	22.8	23.4	+0.6 (−0.4, 1.6)		
Polyunsaturated Fatty Acids (% of total fatty acids)	Placebo	41.6	39.2	−2.5 (−3.6, −1.3)	0.042	0.076^b^
	PRSE	40.9	40.1	−0.8 (−1.9, 0.3)		
Trans Fatty Acids (% of total fatty acids)	Placebo	0.61	0.66	+0.05 (−0.02, 0.11)	0.016	0.048^b^
	PRSE	0.66	0.59	−0.07 (−0.14, −0.01)		

Values are estimated marginal means derived from linear mixed-effects models adjusted for dietary polyphenol intake. Change (Δ) values represent the estimated within-treatment difference between post-treatment and pre-treatment measurements, with 95% confidence intervals (CI). *p*-values correspond to the treatment × time interaction and represent the effect of PRSE relative to placebo. False discovery rate (FDR) adjustment was performed using the Benjamini–Hochberg procedure within predefined outcome families, with *p*-values sharing a superscript letter being part of the same outcome family.

### 3.4 Mood and wellbeing

No significant treatment × time interactions were observed for positive affect, negative affect, or wellbeing index, and these findings remained non-significant following FDR adjustment. Collectively, these findings suggest that PRSE supplementation did not influence mood or wellbeing relative to placebo.

### 3.5 Microbiome

**Figure 3:**
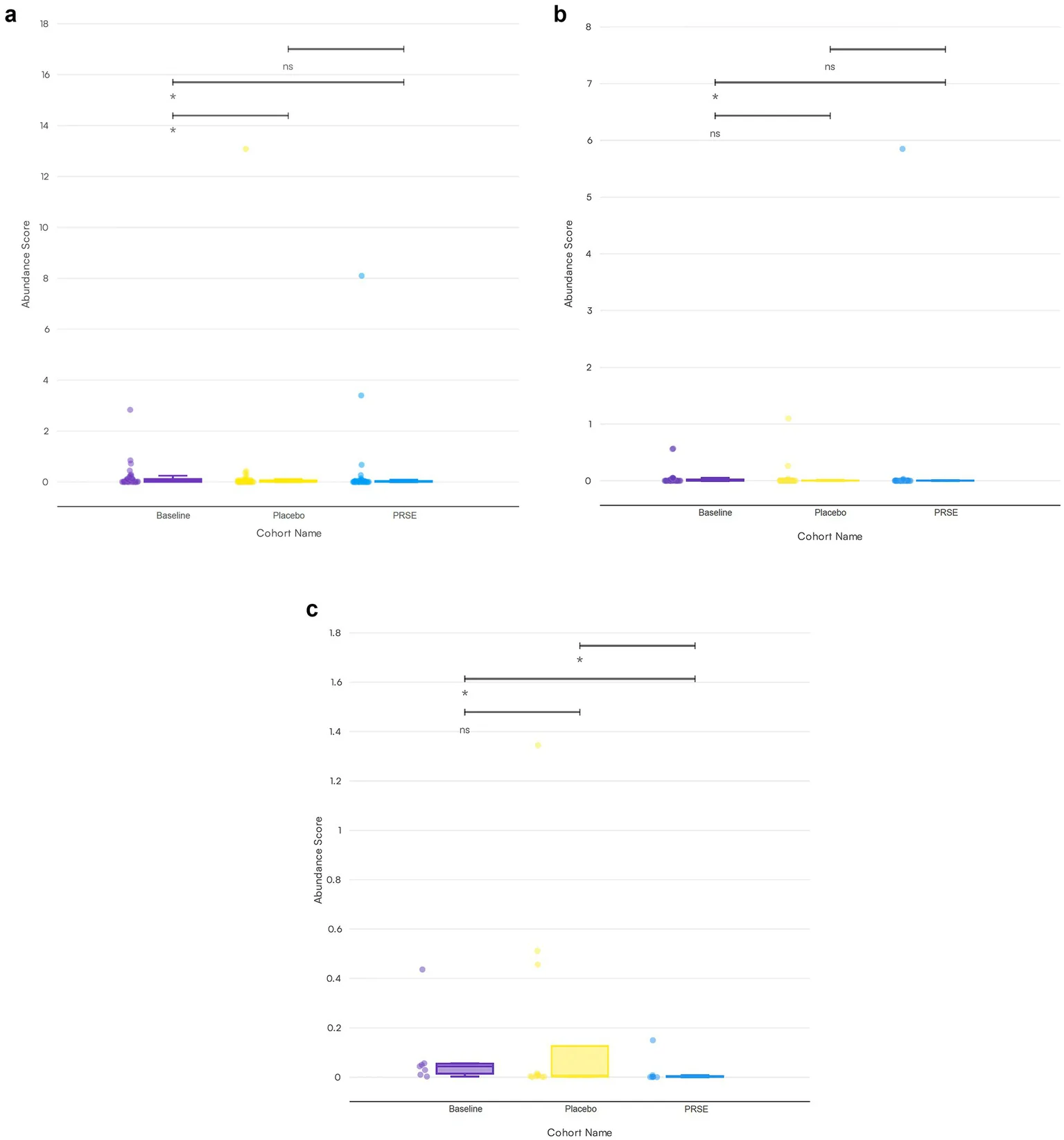
**(a–c)** Measures of abundance distribution by abundance score when comparing the baseline, placebo and PRSE cohorts. P-cresol degradation, pyruvate dehydrogenase and the glycerol III degradation complex are represented by panels **(a–c)**, respectively.

Statistically significant differences were found in selected gut brain and gut metabolic functional pathways (Wilcoxon rank-sum test, *p* < 0.05; Figures 3a–c). Within the gut-brain functions, *p*-cresol degradation abundance scores were significantly lower in both the placebo and PRSE groups compared with baseline; however, no significant difference was detected between the intervention groups (Figure 3a). Similarly, pyruvate dehydrogenase complex abundance scores differed significantly from baseline in both intervention groups but did not differ between placebo and PRSE (Figure 3c).

Among the gut-metabolic modules, glycerol degradation III abundance scores were significantly lower following PRSE supplementation compared with both baseline and placebo, indicating a treatment-specific effect on this microbial metabolic pathway (Figure 3b).

Comparative microbial analyses revealed no significant differences in alpha diversity, beta diversity, or PCoA between baseline, placebo, and PRSE groups when assessing microbial taxa, gut-brain functions, and gut-metabolic functions.

## 4 Discussion

The present study investigated the effects of PRSE supplementation on cardiometabolic biomarkers and gut microbial function in healthy adults. PRSE supplementation was associated with a significantly lower proportion of circulating *trans*-fatty acids, as well as a protective effect against increased VLDL and triglycerides compared to the control. Significant differences were identified in selected microbial functional pathways, without a significant change to the overall microbiome diversity.

### 4.1 Cardiometabolic biomarkers

Among the cardiometabolic biomarkers assessed, PRSE supplementation was associated with selective changes in lipid-related outcomes. Significant treatment × time interactions that remained significant following FDR adjustment were observed for triglycerides, VLDL cholesterol, and *trans*-fatty acids. Although *trans*-fatty acids represent circulating fatty acid species, whereas triglycerides and VLDL cholesterol are markers of triglyceride-rich lipoprotein metabolism, these outcomes are interconnected through hepatic lipid and lipoprotein pathways and may therefore reflect related metabolic responses to PRSE supplementation (23, 24).

Triglyceride and VLDL cholesterol concentrations increased during the placebo period but were lower following PRSE supplementation. This finding is consistent with previous findings where polyphenols reduce fasting triglyceride concentrations, large VLDL particles, and postprandial triglyceride responses, although these effects have primarily been reported in individuals with elevated cardiometabolic risk (25). Moreover, the reduction in circulating *trans*-fatty acids is of particular interest, given that higher circulating concentrations have been associated with inflammation, adverse metabolic outcomes, and increased metabolic risk (26).

Although the underlying mechanisms were not investigated, the observed changes in *trans*-fatty acids, triglycerides, and VLDL cholesterol may reflect related effects on hepatic lipid metabolism. Higher circulating *trans*-fatty acids have been associated with increased triglyceride synthesis, VLDL secretion, and cholesteryl ester transfer protein activity, suggesting a potential link with triglyceride-rich lipoprotein metabolism (27). Moreover, polyphenols may influence lipid metabolism and oxidative balance (10) through AMPK, PPAR, Nrf2, NF-κB, and MAPK signalling pathways (28). Their antioxidant activity may further limit lipid peroxidation and *trans*-fatty acid formation, as supported by *in vitro* evidence showing reduced *trans*-arachidonic acid and thermally induced *trans*-fatty acids following antioxidant exposure (29, 30).

To contextualise the magnitude and potential clinical relevance of these findings, the observed changes were interpreted in relation to associations reported in prospective cardiovascular studies. Specifically, each 10 mg/dL increase in VLDL cholesterol has been associated with an approximately 7% higher risk of incident atherosclerotic cardiovascular disease, independent of traditional cardiovascular risk factors. Although the present study was not designed to evaluate clinical cardiovascular outcomes, these epidemiological associations suggest that the observed changes in VLDL cholesterol may be clinically meaningful if sustained over time (31). In the present study, VLDL cholesterol increased by approximately 8.5 mg/dL during the placebo period but remained essentially unchanged during PRSE supplementation (−1.0 mg/dL). This difference is potentially clinically relevant given that higher VLDL cholesterol has been associated with an increased risk of atherosclerotic cardiovascular disease. Similarly, PRSE supplementation reduced total plasma *trans*-fatty acid content by 0.07%. For context, a prospective study reported that each 0.1% increase in erythrocyte C18:1 *trans*-fatty acid content was associated with a 53% higher prevalence of carotid atherosclerotic plaque after adjustment for traditional cardiovascular risk factors (27). Although direct comparison should be interpreted cautiously because the previous study quantified C18:1 *trans*-fatty acids specifically, whereas the present study measured total *trans*-fatty acids, the observed reduction is consistent with a direction of change associated with a more favourable cardiovascular risk profile (27).

Collectively, these findings suggest that PRSE selectively modulates lipid metabolism, with the most pronounced effects observed for *trans*-fatty acids, triglycerides, and VLDL cholesterol. The detection of these changes in a metabolically healthy cohort indicates that lipid metabolic pathways may remain responsive to polyphenol supplementation even when baseline cardiometabolic markers are within the normal range (32). Although these findings support the biological activity of PRSE, their clinical significance and broader applicability remain to be established. Further studies in populations with elevated cardiometabolic risk are needed to determine whether these metabolic changes translate into meaningful improvements in cardiovascular health outcomes.

### 4.2 Gut microbial functional pathways

While the cardiometabolic findings suggested effects on lipid homeostasis, microbiome analyses provided insight into potential microbial functional changes that may contribute to these responses. The gut microbiome plays a central role in host metabolism and gut–brain communication through the production and transformation of bioactive microbial metabolites.

PRSE supplementation did not significantly alter microbial diversity or overall community composition but was associated with significant differences in selected microbial functional pathways. As these pathways were inferred from shotgun metagenomic sequencing, the findings reflect changes in the predicted functional capacity of the microbial community rather than direct evidence of pathway activity, metabolic flux, or metabolite production. Nevertheless, the results suggest that PRSE may selectively influence microbial functional potential without inducing detectable shifts in overall taxonomic structure. This observation is consistent with emerging evidence that dietary polyphenols can modify microbial functional profiles independently of major changes in community composition, highlighting that alterations in microbial function may precede, or occur in the absence of, measurable taxonomic changes (6).

The most prominent difference following PRSE supplementation was a lower relative abundance of the glycerol degradation III pathway. This pathway encodes microbial glycerol utilisation through the production of dihydroxyacetone phosphate, an intermediate connecting glycerol metabolism with downstream energy-generating pathways (33). In some colonic bacteria, glycerol metabolism contributes to fermentative energy production and redox balance through products such as 1,3-propanediol and acetate (34). The lower pathway abundance may therefore indicate reduced community-level genetic potential for glycerol utilisation. Additionally, differences were observed in pyruvate dehydrogenase complex (PDHC)-related pathways. The PDHC represents a central metabolic node that links glycolysis with Acetyl-CoA production and downstream energy metabolism (35). These differences may reflect variation in the microbial community’s encoded capacity for carbon processing (34).

Differences were also observed in *p*-cresol degradation pathways. P-cresol originates from microbial metabolism of aromatic amino acids and can subsequently be converted by the host to *p*-cresyl sulphate (36), a protein-bound metabolite associated with adverse renal, cardiovascular, and neurological outcomes (37). Several dietary polyphenols, including epi-gallocatechin-3-gallate, proanthocyanidins, and gallic acid, have been reported to reduce *p*-cresol production or inhibit enzymes involved in its formation (38, 39). The observed pathway differences therefore warrant targeted metabolomic investigation to determine whether they correspond to measurable changes in *p*-cresol-related metabolites or host physiology.

This study had several strengths, including its quadruple-blinded, randomised, placebo-controlled crossover design and the multifaceted assessment of metabolic, microbial, and wellbeing outcomes. The inclusion of functional microbiome analyses extended beyond taxonomic profiling alone and provided additional insight into potential host–microbiome interactions associated with PRSE supplementation. Furthermore, the fully remote study design improved accessibility and enabled long-term assessment under free-living conditions.

Several limitations should also be acknowledged. Firstly, participants were generally healthy, and many cardiometabolic biomarkers were likely within normal physiological ranges at baseline, potentially limiting the magnitude of detectable treatment effects (5). The study cohort was predominantly female (36 females and 11 males), which may limit the generalisability of the findings. Sex differences in lipid metabolism, inflammatory responses, gut microbiota composition, and polyphenol metabolism have been reported and may influence the physiological response to PRSE supplementation. Consequently, future studies should aim to determine whether the metabolic and microbial responses to PRSE differ between women and men, including specifically designed to evaluate sex specific responses.

Furthermore, reliance on self-reported measures may have introduced reporting bias and reduced experimental control compared with supervised clinical assessments (40). Although participants were instructed to maintain their habitual diet, dietary intake was assessed primarily through overall diet quality and estimated polyphenol intake. Detailed monitoring of total energy and dietary fat intake was not undertaken. Given the methodological complexity of controlling habitual diet in free-living dietary intervention trials, unmeasured changes in these dietary factors cannot be excluded (41). Adherence was also assessed by self-report, which may have introduced recall or reporting bias (40).

An additional limitation is that microbial functional pathways were inferred from shotgun metagenomic sequencing rather than direct measurement of microbial metabolites. Consequently, the observed pathway differences should be interpreted as changes in predicted functional potential rather than confirmed metabolic activity. Targeted metabolomic or transcriptomic analyses are required to establish the biological consequences of these findings (42).

Finally, the crossover design carries a risk of residual carryover effects between intervention periods, which cannot be completely excluded. In order to reduce the risk of carryover effects between intervention arms, each intervention period was extended to 90 days, allowing sufficient time for intervention effects to develop and stabilise before outcome assessment. In addition, phase, sequence, and sequence-by-treatment interaction terms were included in the statistical models to assess and account for potential carryover effects. Although these design and analytical strategies strengthen the validity of the findings, they cannot definitively exclude residual carryover, and this should be considered when interpreting the results (15, 43).

The potential impact of carryover is likely to differ across outcomes. The significant treatment effects observed for circulating triglycerides and VLDL cholesterol involve biomarkers with relatively rapid turnover. VLDL particles have a residence time of approximately 2–4 days (44), while triglycerides are continuously metabolised and turned over at a rate of approximately 13–14 mg/kg/h (45). These kinetics may reduce the likelihood of prolonged direct persistence of altered circulating triglyceride and VLDL cholesterol concentrations after supplementation ceased; however, they do not exclude persistent effects on the biological pathways regulating lipid metabolism (15).

Greater uncertainty applies to outcomes with longer accumulation or adaptation periods, including HbA1c, lipidomic profiles, and gut microbiome composition. Previous dietary intervention studies have demonstrated that microbiome responses to substantial changes in macronutrient intake may develop over several weeks and can follow *non*-linear trajectories (46, 47). However, these interventions involved major dietary changes, whereas the present study evaluated a defined supplement without intentionally altering participants’ habitual diets; therefore, their adaptation timelines cannot be directly extrapolated to PRSE supplementation. Nevertheless, because the persistence of PRSE-related effects is unknown, findings should be interpreted with greater caution. Future studies targeting these outcomes should consider a parallel-group design or an outcome-specific washout period.

## 5 Conclusion

In this cohort, PRSE supplementation produced selective effects on lipid-related biomarkers, including a reduction in the circulating proportion of trans-fatty acids and attenuation of increases in triglyceride and VLDL cholesterol concentrations. These findings suggest that polyphenol-rich interventions may influence lipid homeostasis even when baseline cardiometabolic markers are largely within normal range.

PRSE supplementation was also associated with differences in selected microbial functional pathways without altering overall microbial diversity or community composition. These findings suggest changes in microbial functional potential rather than broad taxonomic shifts or confirmed changes in microbial metabolic activity (48).

Taken together, these findings demonstrate the biological activity of PRSE, with consistent effects on lipid-related biomarkers and gut microbial functional capacity. Although the magnitude of these changes was modest, they indicate that PRSE can modulate key metabolic pathways even in a relatively young, metabolically healthy population with limited scope for physiological improvement. Consequently, the observed effects may underestimate the potential benefits in individuals with impaired cardiometabolic health. Nevertheless, these findings should not be extrapolated to populations with obesity, metabolic syndrome, dyslipidaemia, or diabetes without further investigation. Larger, adequately powered clinical trials incorporating targeted metabolomic profiling and mechanistic assessments in populations with elevated cardiometabolic risk are warranted to determine whether these metabolic adaptations translate into clinically meaningful improvements in cardiovascular and metabolic health.

## Data Availability

Data described in the manuscript, code book, and analytic code will be made available upon request pending approval. The data presented in this study has been deposited to the National Center for Biotechnology Information (NCBI) Bioproject database under accession number http://www.ncbi.nlm.nih.gov/bioproject/1499345 (PRJNA149934).
